# Differential Effects of Methoxy Group on the Interaction of Curcuminoids with Two Major Ligand Binding Sites of Human Serum Albumin

**DOI:** 10.1371/journal.pone.0087919

**Published:** 2014-02-03

**Authors:** Hiroki Sato, Victor Tuan Giam Chuang, Keishi Yamasaki, Noriyuki Yamaotsu, Hiroshi Watanabe, Kohei Nagumo, Makoto Anraku, Daisuke Kadowaki, Yu Ishima, Shuichi Hirono, Masaki Otagiri, Toru Maruyama

**Affiliations:** 1 Department of Biopharmaceutics, Graduate School of Pharmaceutical Sciences, Center for Clinical Pharmaceutical Science, Kumamoto University, Kumamoto, Japan; 2 School of Pharmacy, Curtin Health Innovation Research Institute, Curtin University, Perth, Australia; 3 Faculty of Pharmaceutical Sciences, DDS Research Institute, Sojo University, Kumamoto, Japan; 4 School of Pharmacy, Kitasato University, Tokyo, Japan; University of Hyderabad, India

## Abstract

Curcuminoids are a group of compounds with a similar chemical backbone structure but containing different numbers of methoxy groups that have therapeutic potential due to their anti-inflammatory and anti-oxidant properties. They mainly bind to albumin in plasma. These findings influence their body disposition and biological activities. Spectroscopic analysis using site specific probes on human serum albumin (HSA) clearly indicated that curcumin (Cur), demethylcurcumin (Dmc) and bisdemethoxycurcumin (Bdmc) bind to both Site I (sub-site Ia and Ib) and Site II on HSA. At pH 7.4, the binding constants for Site I were relatively comparable between curcuminoids, while the binding constants for Site II at pH 7.4 were increased in order Cur < Dmc < Bdmc. Binding experiments using HSA mutants showed that Trp214 and Arg218 at Site I, and Tyr411 and Arg410 at Site II are involved in the binding of curcuminoids. The molecular docking of all curcuminoids to the Site I pocket showed that curcuminoids stacked with Phe211 and Trp214, and interacted with hydrophobic and aromatic amino acid residues. In contrast, each curcuminoid interacted with Site II in a different manner depending whether a methoxy group was present or absent. A detailed analysis of curcuminoids-albumin interactions would provide valuable information in terms of understanding the pharmacokinetics and the biological activities of this class of compounds.

## Introduction

**Figure 1:**
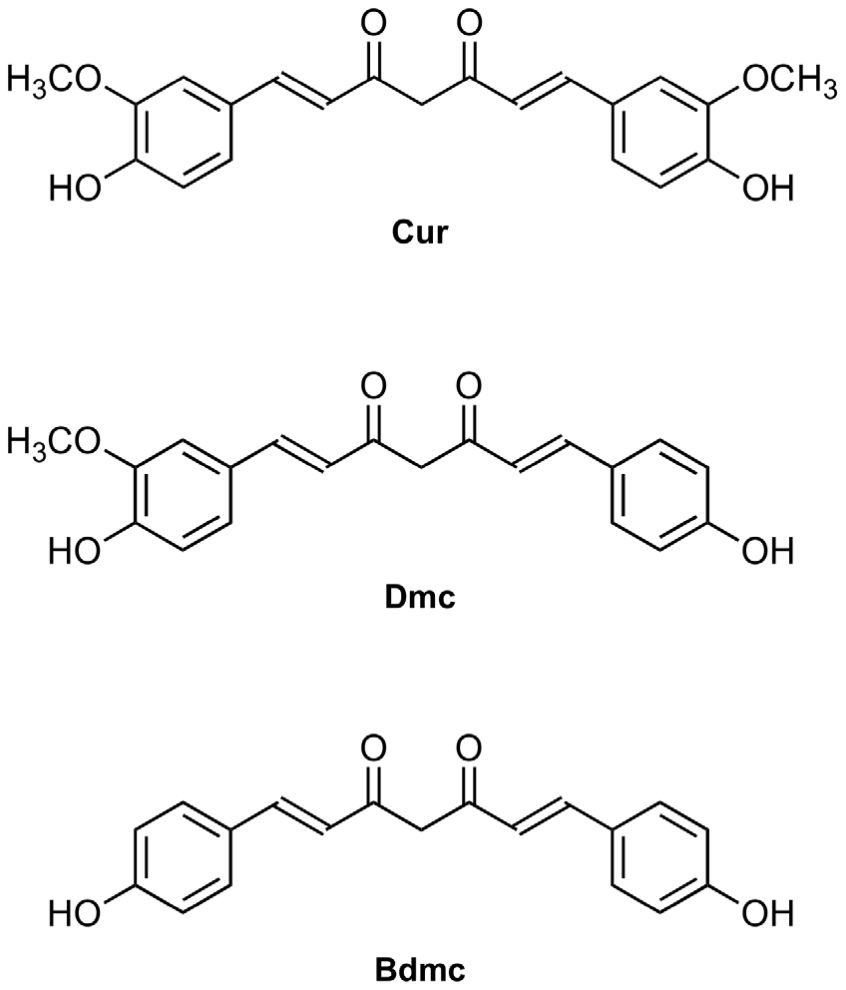
Structures of curcumin (Cur), demethoxycurcumin (Dmc), and bisdemethoxycurcumin (Bdmc).

In recent years, active investigations have been conducted regarding clinical applications of curcumin (Cur), a pigment component of Turmeric (Curcuma longa L.) and rhizomatous herbaceous perennial plant of the ginger family [1]. The scientific research and clinical trials conducted so far were not specifically focussed exclusively on Cur only, but were on "curcuminoids", a mixture of Cur analogues such as demethoxycurcumin (Dmc) or bisdemethoxycurcumin (Bdmc). Curcuminoids have similar chemical structures except for the number of methoxy groups on each of the two phenyl moieties. Cur contains one methoxy group on each of its two phenyl moieties, Dmc contains one methoxy group on one phenyl moiety, whereas Bdmc is devoid of any methoxy groups (Figure 1). Curcuminoids have been found to possess a variety of biologically activities such as anti-inflammatory, anti-oxidant, anti-cancer, anti-virus, anti-infective, anti-malarial and wound healing [2], [3]. Furthermore, interesting structure-activity relationship findings have been reported for the curcuminoids. For example, Cur shows the highest anti-oxidant and anti-inflammatory effects [4], [5] whereas Dmc and Bdmc are superior to Cur in inhibiting cancer cell proliferation [6], and for improving the clearance of amyloid β (Aβ) in an animal model with a congenital immune deficiency [7]. Research on the therapeutic effects of curcuminoids in treating Alzheimer's disease (AD) has been actively carried out. Curcuminoids have been reported to suppress the accumulation of the Aβ protein in the brain of an AD mouse model, reduce the formation of amyloid plaques [8] and improve the impaired Aβ clearance due to an innate immunity deficiency [7]. Therefore, curcuminoids have considerable potential for use as therapeutic agents for the clinical treatment or prevention of AD.

Human serum albumin (HSA), is a monomeric protein consisting of 585 amino acid residues, with a molecular weight of approximately 66,500 Da [9]. HSA is a plasma protein that functions as a carrier for various endogenous and exogenous substances [10], [11]. Two major ligand binding sites of HSA, site I and site II [12], were reported to be located in subdomain IIA and subdomain IIIA respectively [13], [14]. The distribution and pharmacological actions of curcuminoids are controlled by their strong binding to HSA in the systemic circulation after oral absorption. Therefore, clarification of the binding mechanism and the nature of the interactions of curcuminoids with HSA are essential in predicting the pharmacokinetics and therapeutic effects of curcuminoids and their potential interactions with other pharmaceutical agents. Researches on the interaction of curcuminoids with HSA have, so far, mostly focused on Cur. For example, Reddy et al. reported that Cur binds to HSA at physiological pH and there are at least two Cur binding sites on HSA [15]. In addition, Zsila et al. using displacement experiments aided by spectroscopic techniques found that one of the binding sites of Cur is site I [16]. However, the other binding site of Cur on HSA has not been determined, and the binding sites of Bdmc and Dmc on HSA remain unidentified.

In this study, site directed mutagenesis and spectroscopic techniques were used to evaluate the molecular mechanism of the binding properties and binding sites of curcuminoids on HSA, with emphasis on the role of the methoxy group of curcuminoids in binding to HSA. Furthermore, computer modeling of the HSA-curcuminoids complex was performed to further evaluate the mechanisms of binding at the molecular level and the results were compared to the other experimental findings obtained in this study.

## Materials and Methods

### Materials

HSA was donated by the Chemo-Sera-Therapeutic Research Institute (Kumamoto, Japan). Warfarin potassium (WF) (Eisai Co., Tokyo, Japan), phenylbutazone (PBZ) (Ciba-Geigy, Summit, NJ, USA), ibuprofen (IBF) (Sanwakagaku Co., Tokyo, Japan), diazepam (DZP) (Nippon Roche K. K., Tokyo, Japan), dansylsarcosine (DNSS) and iodipamide (IDP) (Sigma, St. Louis, MO, USA) were obtained as pure substances from the manufacturers. Cur, Dmc, Bdmc, and sodium caprylate (CAP) were purchased from Nacalai Tesque (Kyoto, Japan). All other reagents were of analytical grade. Curcuminoids were dissolved in 100% ethanol.

### Defatting of Albumin

HSA was defatted with activated charcoal using the method by Chen with some modifications as described previously [17]. HSA molecular weight was assumed to be 66,500 Da when calculating the molar concentrations.

### Production of Oxidized HSAs

To prepare metal-catalyzed oxidation-HSA (MCO-HSA), HSA (300 µM) was incubated in 0.067 M sodium phosphate buffer (pH 7.4) at 37°C in an oxygen-saturated solution containing sodium ascorbate (100 mM) and FeCl_2_ (10 µM) [18]. Ascorbate was added to reduce the Fe^3+^ formed in the oxidation back to Fe^2+^. Aliquots were withdrawn after different time intervals (12, 24 h), and the oxidative process was terminated by cooling and removing the oxidants by extensive dialysis against water. The MCO-HSAs and HSA were stored at −20°C until used.

### Production and Purification of Recombinant Wild-Type HSA and W214A, R218A, R410A, Y411A mutants

Recombinant wild-type HSA (WT-HSA) and single-residue mutants of W214A, R218A, R410A and Y411A were produced following the recombinant DNA techniques essentially described by Watanabe et al [19], [20]. WT-HSA and the HSA mutants were stored at −20°C until used.

### Fluorescence Quenching Experiments

Fluorescence quenching experiments were carried out using a Jasco FP-770 fluorescence spectrometer (Tokyo, Japan), with thermostatically controlled devices and 5 nm excitation and emission bandwidths. Emission spectra in the absence and presence of curcuminoids were recorded at 300–400 nm. Fluorescence titration was performed by keeping the HSA concentration (2 µM) constant and stoichiometrically varying the curcuminoids concentration (0–12 µM). Measurements were made using a 10 mm path length quartz cells with the sample in 0.067 M phosphate buffer pH 6.0 and 0.1 M Tris/HCl buffer pH 7.4 and 9.0.

The binding constants of curcuminoids were calculated from fluorescence quenching experimental data. The Trp residue at position 214 on HSA was excited at 295 nm and the intensity of the emission was monitored at 362 nm in the presence of curcuminoids. Data were analyzed using the modified Stern-Volmer equation as shown in the following equation:
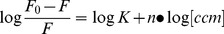
(1)


where n is the slope (i.e. the number of binding sites), K is the binding constant and [ccm] is the concentration of curcuminoids. F_0_ and F are the fluorescence intensities in the absence and presence of curcuminoids.

### Fluorescent Probe Displacement Experiments

WF and DNSS were used as the fluorescent probes for site I and site II, respectively [21]. Both probes were dissolved in 100% ethanol. The displacement experiments of WF and DNSS by curucuminoids were performed by recording the fluorescence of a solution containing 0.5 µM WF (or 1 µM DNSS) and 10 µM HSA while gradually increasing the curucuminoids concentration from 0 to 4 µM. The excitation wavelengths for WF and DNSS were 320 and 350 nm, respectively. The emission spectra for WF and DNSS were recorded in the range of 350–450 nm, 400–600 nm, respectively.

The binding constants of curcuminoids (K_ccm_) were determined based on data from the displacement experiments. The binding constants of curcuminoids (K_ccm_) were calculated from the following equation that takes into account the competitive displacement between the probe and curcuminoids [22]. 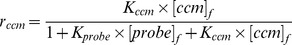
(2)


In this equation, r_ccm_ denotes the concentration of bound curcuminoids ([ccm]_b_) relative to the total protein ([P_t_]) and [ccm]_f_ is the concentration of free curcuminoids. The binding constants of fluorescent probes (K_probe_) were calculated using the method described by Sudlow [23]. The K_probe_ of warfarin at pH 7.4 and pH 9.0 were 3.4×10^5^ and 7.1×10^5^ M^−1^, respectively, whereas the K_probe_ of DNSS at pH 7.4 and pH 9.0 were 7.0×10^5^ and 6.8×10^5^ M^−1^, respectively. In this analysis, the number of binding sites for both probes was found to be 1 at pH 7.4 and pH 9.0. [ccm]_f_ and [ccm]_b_ was also estimated by the degree of displacement. The concentration of free probe ([probe]_f_) was determined by subtracting the concentration of bound probe ([probe]_b_) from the total concentration of the probe ([probe]_t_). The bound concentration of the fluorescent probe in the absence of curcuminoids ([probe]_b_^initial^) was calculated by the following equation: 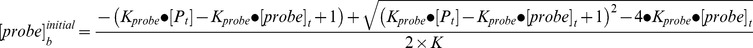
(3)


Therefore, [probe]_f_ in the presence of curcuminoids, was represented using fluorescence intensity in the absence and presence of curcuminoids (F_initial_ and F).
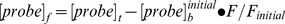
(4)


The linear correlation was first established for the warfarin-curcuminoids system (competitive interaction at primary site) so that similar data to that shown in Table 1 could be obtained. The secondary site binding constants of curcuminoids were determined from data of DNSS-curcuminoids system. In this analysis, a linear correlation established for the warfarin-curcuminoids system at pH 9.0 was also used to determine the binding constant of the curcuminoids to their secondary sites, since the binding constants of DNSS at pH 7.4 and pH 9.0 are consistent with that of warfarin at pH 9.0.

**Table 1:** Number of the binding sites (n) and binding constants (K) of curcuminoids to Site I on HSA at different pH, determined by fluorescence quenching experiments.

	Cur		Dmc		Bdmc	
	n	K (10^5^ M^−1^)	n	K (10^5^ M^−1^)	n	K (10^5^ M^−1^)
pH 6.0	1.07	3.11±0.06	1.08	2.79±0.02	1.11	1.75±0.03
pH 7.4	1.09	2.52±0.04	1.28	2.28±0.05	1.23	1.77±0.03
pH 9.0	1.05	2.99±0.09	1.04	3.28±0.07	1.06	2.73±0.06

The data are average values of five experiments (± S.D.).

### Circular Dichroism (CD) Spectroscopy

CD spectra were recorded between 350 and 600 nm on a Jasco J-820 spectropolarimeter (Tokyo, Japan) in a cuvette with a 1 cm path length. All spectra were accumulated three times with a bandwidth of 1.0 nm and a resolution of 0.5 nm at a scan speed of 100 nm/min. Induced CD is defined as the CD of the curcuminoids-HSA mixture minus the CD of HSA alone at the same wavelengths and is expressed as ellipticity in millidegrees (mdeg).

### CD Displacement Experiments

CD displacement measurements were performed in 0.1 M Tris-HCl buffer (pH 9.0 and 37°C). WF, PBZ and IDP were used as the site I marker ligands [22], whereas IBF, CAP and DZP were used as the site II marker ligands [24]. The displacement experiments were performed by recording the CD value (at 427 nm) of a 25 µM curcuminoid solution and 50 µM HSA with a displacer (site marker ligands)-to-curcuminoids molar ratio increasing from 0 to 6.0.

### Evaluation of the Binding Property of Curcuminoids to Oxidized HSA

The binding of curcuminoids (25 µM) to HSA and oxidized HSAs (50 µM) in Tris-HCl buffer (pH 9.0 and 25°C) was studied by CD measurement.

### Evaluation of the Binding Property of Curcuminoids to Mutant HSAs

The binding of curcuminoids (25 µM) to WT-HSA and the HSA mutants (50 µM) in Tris-HCl buffer (pH 9.0 and 25°C) was studied by CD measurement.

### Docking Simulation of Curcuminoids to Sites I and II of HSA

In order to demonstrate the difference between the bindings of Cur, Dmc and Bdmc, we attempted to dock each compound into the two major ligand binding sites of HSA (sites I and II). The four crystal structures of HSA were taken from the RCSB Protein Data Bank as the docking template structures (PDB IDs: 1h9z and 2bxq for site I, 2bxf and 2bxg for site II). Using Prime 1.6 in the Schrödinger Suite (Schrödinger, LLC., Portland, OR, USA), the missing atoms in the PDB structures were modeled. The ionization states of the curcuminoids were determined based on pK_a_ calculations (ADMET Predictor 4.0, Simulations Plus, Inc., Lancaster, CA, USA). All docked ligands were prepared by LigPrep 2.3 in the Schrödinger Suite. The standard precision of Glide 5.0 in the Schrödinger Suite was used as the docking program. The top-1 poses of GlideScore were chosen as the binding poses [25]. When test ligands for site I (WF, PBZ and IDP) and for site II (IBF and DZP) were used, the above settings reproduced the correct binding modes within 2Å form the X-ray structures (1h9z of *R*-WF, 1ha2 of *S*-WF, 2bxq of PBZ, 2bxn of IDP, 2bxg of IBF and 2bxf of DZP). The docking calculations were performed using 28 nodes of a Dell PowerEdge 1950III (Quad Core Xeon X5460, 3.16 GHz, 56 CPUs in total). Molecular graphics were generated by SYBYL 8.1 (Tripos, L.P., St. Louis, MO, USA) on an HP xw8400 (Dual Core Xeon 5160; 3.00 GHz; 2 CPUs).

## Results

### Fluorescence Quenching Experiments

HSA contains a Trp residue at position 214 which is in the vicinity of site I, hence it is possible to assess the ligand binding of HSA to site I via quantitatively monitoring the quenching of the Trp214 residue intrinsic fluorescence in the presence of various ligand concentrations. When the fluorescence intensity of Trp214 was monitored at various curcuminoids concentrations, a concentration-dependent fluorescence quenching was observed for all curcuminoids examined. The binding parameters can be calculated using the modified Stern-Volmer equation as shown in Equation (1) (Table 1).

The estimated number of binding sites at pH 7.4 on HSA was 1.0 for all of the curcuminoids examined. In addition, the binding constants for Cur, Dmc, and Bdmc were 2.52×10^5^ M^−1^, 2.28×10^5^ M^−1^, 1.77×10^5^ M^−1^, respectively. The binding parameters for Cur estimated in this study was in agreement with the previously reported values [15], [34]. When the experiments were repeated at pH 6.0 and pH 9.0, no significant change in the binding parameters was observed. The binding of curcuminoids to HSA was not affected by changes in pH, and the binding ability (nK value) for the curcuminoids to HSA did not differ greatly. In other words, the results suggest that the binding of curcuminoids is less likely to be affected by the N-B transition of HSA, which refers to a conformational change in HSA in response to a shift from neutral to alkaline pH.

### Fluorescent Probe Displacement Experiments

**Figure 2:**
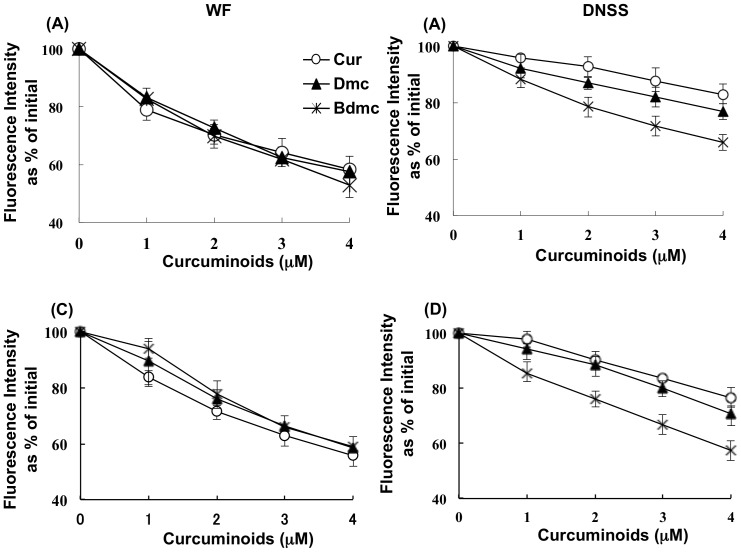
Effects of curcuminoids on the fluorescent intensity at pH(A) and DNSS (B) bound to HSA; the experiment was repeated at pH 9.0 for WA (C) and DNSS (D). Displacement experiments for WF and DNSS by curucuminoids were performed by recording the fluorescence of a solution containing 0.5 µM WF (or 1 µM DNSS) and 10 µM HSA while gradually increasing the curucuminoid concentration from 0 to 4 µM. The excitation wavelengths for WF and DNSS were 320 and 350 nm, respectively. The emission spectra for WF and DNSS were recorded in the range of 350–450 nm, 400–600 nm, respectively. Results are the means ± SD of triplicate experiments. *, **, ***P< 0.05 versus Cur.

In order to determine the binding site of curcuminoids on HSA, site specific fluorescent probes displacement experiments were performed using WF and DNSS as site I and site II specific fluorescent probes, respectively at pH 7.4. The displacement experiments were repeated at pH 9.0. Firstly, at pH 7.4, the addition of curcuminoids caused a concentration-dependent reduction in the WF fluorescence intensity, with 4 µM of curcuminoids causing a reduction of about 60% of the initial fluorescence intensity (Figure 2A). No significant difference in the degree of quenching was observed for the three curcuminoids examined. On the other hand, although curcuminoids also caused a reduction of the DNSS fluorescence intensity in a concentration-dependent manner, the degree of quenching varied, with the largest effect for Bdmc, followed by Dmc and then Cur (Figure 2B). Based on the displacement data for WF, the binding constants of Cur, Dmc and Bdmc for site I at pH 7.4 were determined to be 2.54±0.31×10^5^ M^−1^, 2.46±0.25×10^5^ M^−1^ and 2.24±0.25×10^5^ M^−1^, respectively. (Table 2) These are consistent with data obtained in the fluorescence quenching experiments. (Table 1) Similarly, the binding constants of Cur, Dmc and Bdmc for site II at pH 7.4 were determined to be 2.04±0.21×10^4^ M^−1^, 3.83±0.20×10^4^ M^−1^ and 7.56±1.00×10^4^ M^−1^, respectively. (Table 2) Similar results were obtained when the experiments were repeated at pH 9.0. (Figure 2C, D, Table 2)

**Table 2:** Binding constants (K) of curcuminoids to Sites I and II on HSA at pH 7.4 and pH 9.0, determined by fluorescent probe displacement experiments.

	Site I			Site II		
	K (10^5^ M^−1^)			K (10^4^ M^−1^)		
	Cur	Dmc	Bdmc	Cur	Dmc	Bdmc
pH 7.4	2.54±0.31	2.46±0.25	2.24±0.44	2.04±0.21	3.83±0.20	7.56±1.00
pH 9.0	3.11±0.52	2.98±0.39	2.71±0.22	2.69±0.46	4.07±0.51	11.3±0.55

The data are average values of five experiments (± S.D.).

### Impact of Site Marker Ligands on the Induced CD Spectra of HSA-Curcuminoids Complexes

**Figure 3:**
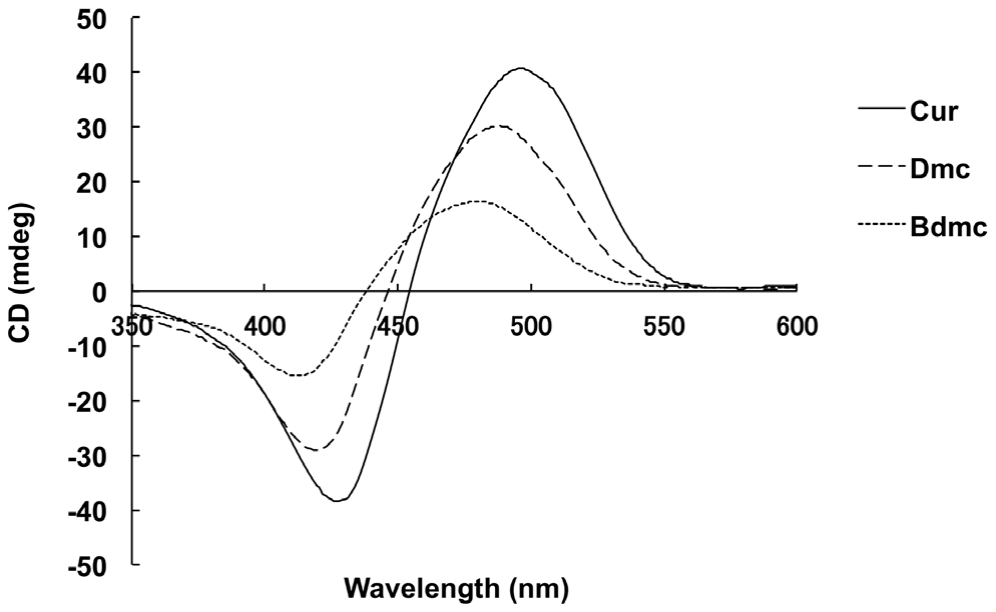
Induced CD spectra of curcuminoids-HSA systems at pH 9.0. Curcuminoids (30 µM) were added to HSA (30 µM) in Tris-HCl buffer (pH 9.0 and 25°C). CD spectra were recorded between 350 and 600 nm on a Jasco J-820 spectropolarimeter (Tokyo, Japan) in a cuvette with 1 cm pathlength. All spectra were accumulated three times with a bandwidth of 1.0 nm and a resolution of 0.5 nm at a scan speed of 100 nm/min.

Cur alone does not show any CD because it is optically inactive. However, when it is bound to HSA in an asymmetric environment of the binding site, Cur will show an induced-CD spectra [16]. At physiological pH of 7.4 the intensity of this induced-CD is weak, but under alkaline conditions, Cur-HSA shows strong bisignate CD bands due to the exciton coupling of the two feruloyl chromophores [15]. In the present study, the HSA-curcuminoids CD spectrameasured at pH 9.0 were consistent with those previously reported [16], induced biphasic Cotton effects associated with the HSA binding of Cur were observed. (Figure 3) Similar CD spectra were also observed for Bdmc and Dmc. The induced biphasic Cotton effects took a similar pattern of emergence from positive to negative for all curcuminoids, suggesting that curcuminoids were in a right-handed conformation when bound to HSA [26].

**Figure 4:**
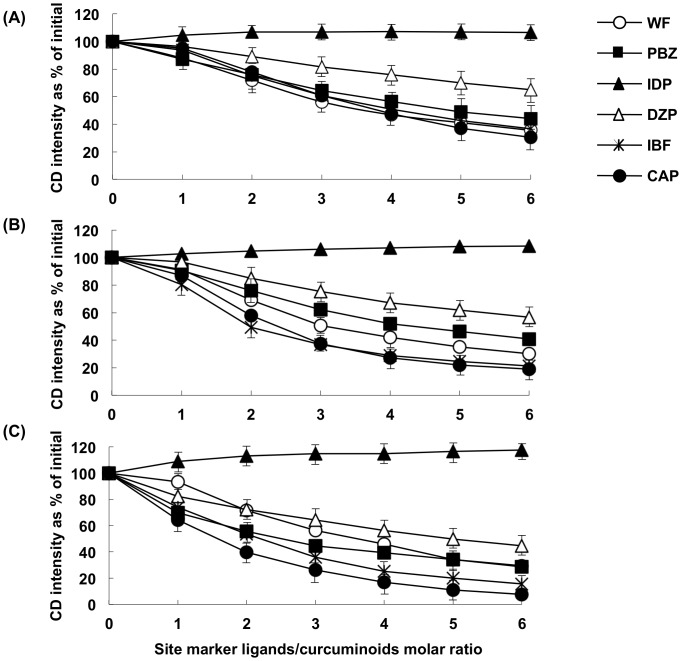
Effects of site marker ligands on the intensity of induced CD of curcuminoids- HSA systems (A: Cur; B: Dmc; C: Bdmc). CD displacement measurements were performed in 0.1-HCl buffer (pH 9.0 and 37°C). WF, PBZ and IDP were used as the site I marker ligands, whereas IBF, CAP and DZP were used as the site II marker ligands. The displacement experiments were performed by recording the CD value (at 427 nm) of a solution containing 25 µM curcuminoids and 50 µM HSA with displacer (site marker ligands)-to-curcuminoids molar ratio increasing from 0 to 6.0.

We further investigated the influence of the site specific marker ligands on the CD spectrum derived from each HSA-curcuminoid complexes at pH 9.0. In this study, WF, PBZ, and IDP were used as site I markers, while DZP, IBF, and CAP were used as site II markers. The relative CD intensity of Cur-HSA was significantly reduced by WF and PBZ to about up to 36% and 44%, respectively, but the CD intensity increased slightly in the presence of IDP. On the other hand, the CD intensity was attenuated in the order DZP, IBF, and CAP, corresponding to a reduction of 65%, 37%, and 30% respectively (Figure 4A). Similar trends were observed with Dmc and Bdmc but Dmc and Bdmc caused a greater decrease in the CD intensity than Cur (Figure 4B, C). This could be due to differences in their binding affinities to site II because, as mentioned above, the binding constants of Dmc and Bdmc to site II were larger than that of Cur (Table 2).

### Effect of Metal-Catalyzed Oxidation on the Binding Properties of HSA for Curcuminoids

**Figure 5:**
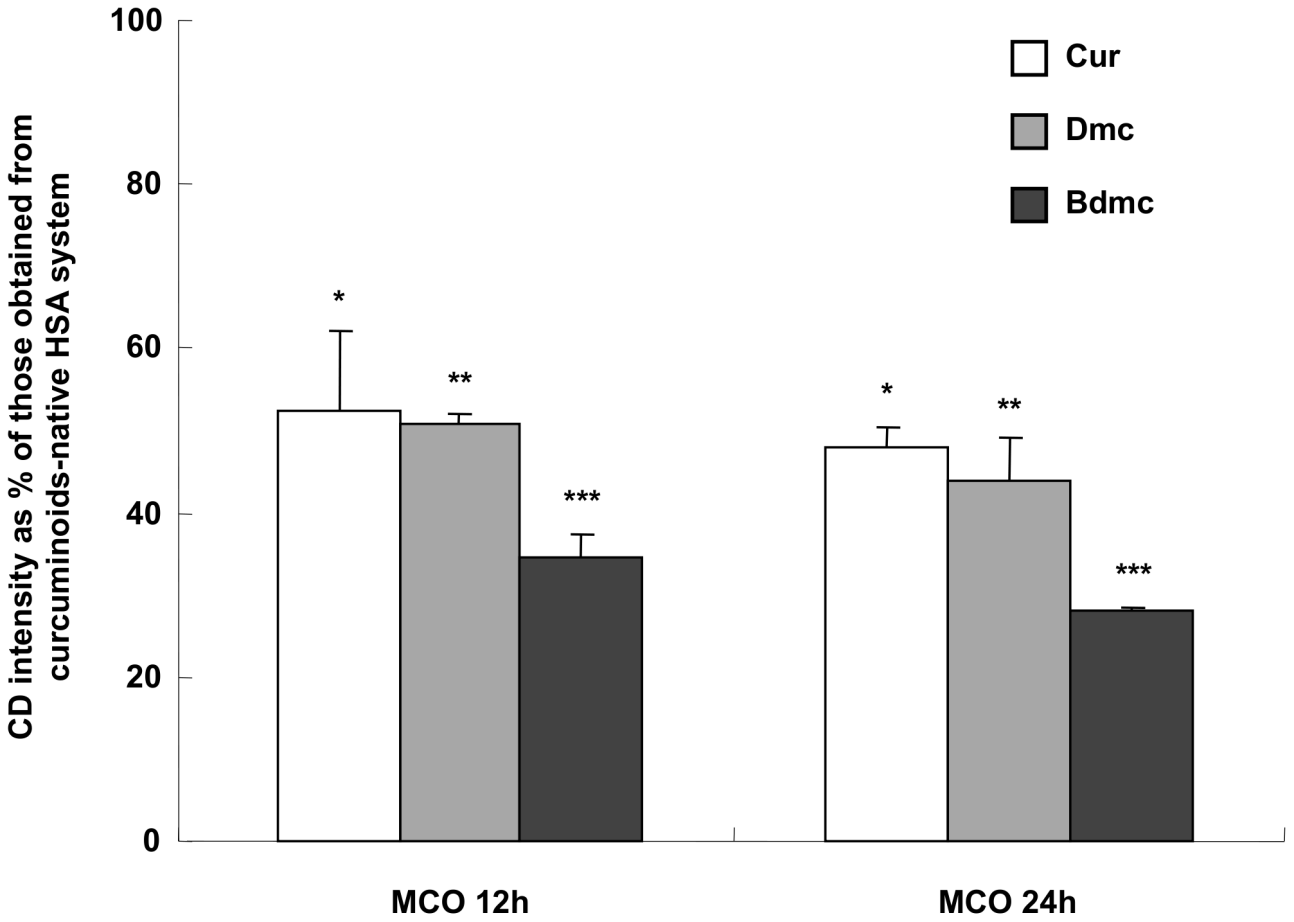
Effects of metal catalized oxidation on the intensity of CD induced of curcuminoids-HSA systems. The binding of curcuminoids (25 µM) to HSA and oxidized HSAs (50 µM) in Tris-HCl buffer (pH 9.0 and 25°C) was studied by CD measurement. Results are the means ± SD of triplicate experiments. *, **, ***P< 0.05 versus native HSA.

We have reported previously that oxidation of HSA via metal-catalyzed oxidation (MCO) did not affect the ligand binding to site I, but binding to site II was reduced significantly [27]. When the binding of three curcuminoids to unmodified HSA and oxidised HSA derived from various MCO reaction times was compared, a significant decrease in CD intensity was observed depending on the degree of MCO for all of the curcuminoids. (Figure 5) This strongly suggests that curcuminoids bind to site II. In addition, a comparison of the CD intensity of HSA treated by MCO for 24 h shows a decrease in the CD intensity in the order of Bdmc> Dmc> Cur. From this interesting result, it appears that each curcuminoid interacted with site II in a different manner with different individual binding capacity can be inferred.

### Evaluation of Curcuminoids Binding to HSA Mutants

**Figure 6:**
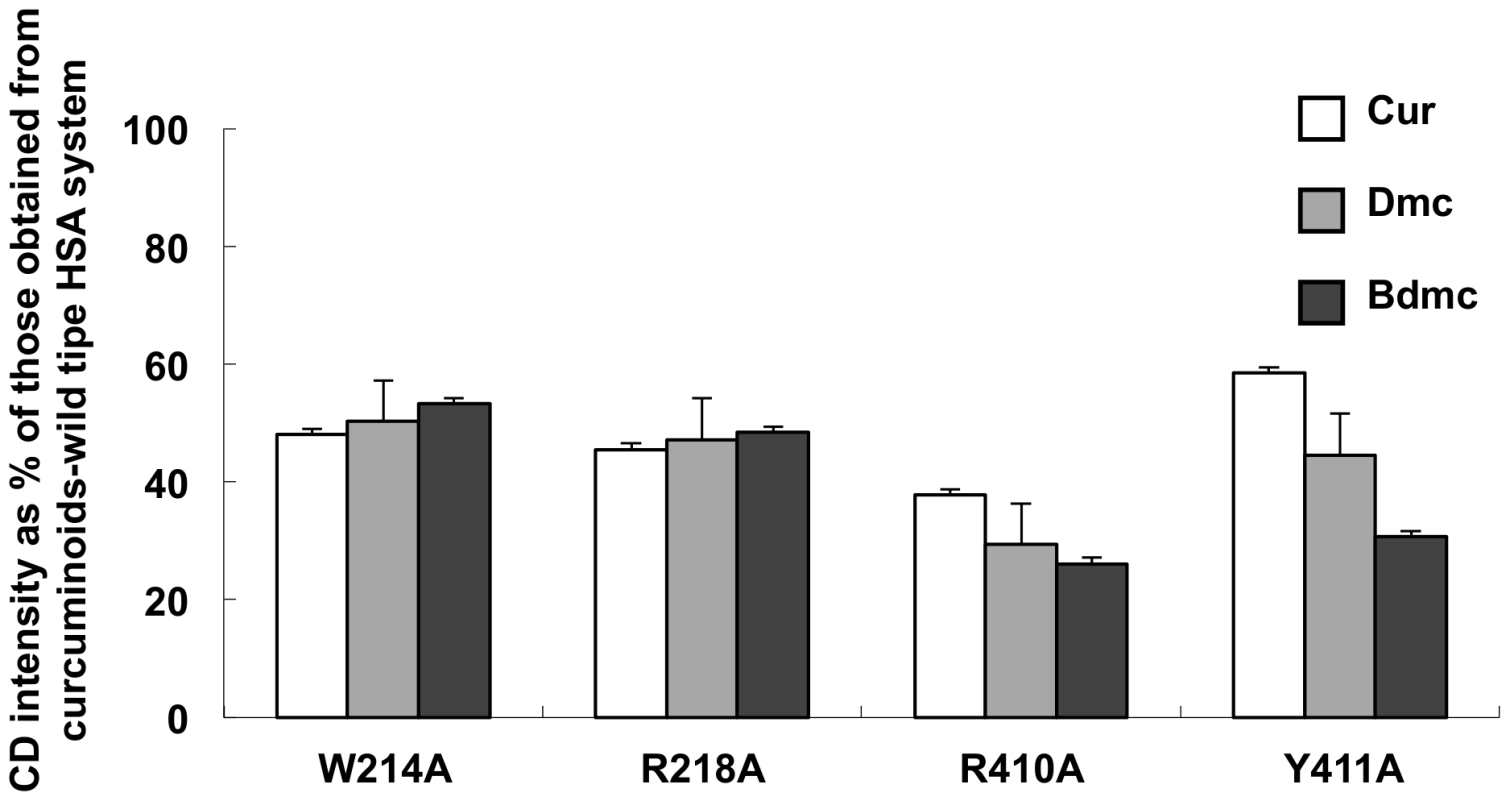
Effects of HSA mutation on the intensity of induced CD of curcuminoids-HSA systems at pH 9.0. The binding of curcuminoids (25 µM) to WT-HSA and HSA mutants (50 µM) in Tris-HCl buffer (pH 9.0 and 25°C) was studied by CD measurement. Results are the means ± SD of triplicate experiments. *, **, ***P< 0.05 versus native HSA.

Site-directed mutagenesis has been carried out to identify the amino acid residues that involved in the binding of Curcuminoids to HSA. Amino acid residues at sites I and II that are known to be involved in ligand binding [13] were replaced with alanine to produce mutants of site I (W214A and R218A), and site II (Y411A and R410A) for curcuminoids binding studies. The CD intensity for the complexes of W214A with Cur, Dmc, and Bdmc was 48, 50, 53% of that of wild type HSA respectively, whereas for R218A were 46, 47, 48% of that of the wild type HSA (Figure 6). On the other hand, the CD intensity for the complexes of R410A was about 38, 29, 26%, and Y411A were 58, 45, 31% that of the wild type curcuminoid-HSA complexes, respectively. Regarding the previous finding that these mutations did not significantly influence the structure of site I and II, at least the four mutated residues appear to play a role in the binding of curcuminoids to HSA. For the site I mutants, R218A and W214A, the extent of decrease in binding was almost the same among all of three curcuminoids (Cur  =  Dmc  =  Bdmc). In contrast, for the site II mutants, Y411A and R410A, the level of decrease in binding varies in the order of Bdmc> Dmc> Cur.

### Docking Simulation of Curcuminoids to Sites I and II of HSA

Based on the estimated pKa for curcuminoids, it is highly possible that Bdmc and Dmc carry one negative charge and Cur carries two negative charges at pH 9.0. The binding affinity of curcuminoids in this state for sites I and II was then predicted by the Glide SP Score. The Glide SP Score for site I were –7.79 (Cur), –7.88 (Dmc), and –7.80 (Bdmc), the maximum difference was 0.09. This indicates that there was no major difference in the binding affinity of each curcuminoid for site I. On the other hand, the Glide SP Score for site II decreased in the order of Bdmc<Dmc<Cur. A difference of about 0.14 was found between Dmc and Cur, or between Dmc and Bdmc, suggesting that the binding affinity of curcuminoids for the site II is different and decreases following the order of Bdmc> Dmc> Cur.

**Figure 7:**
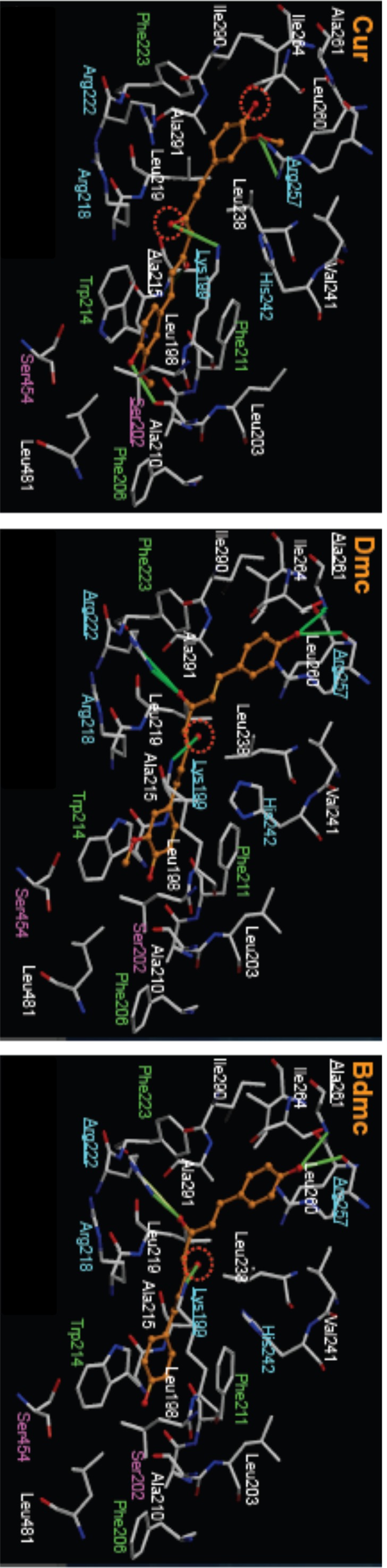
Molecular docking of curcuminoids in site I of HSA. The optimized docking poses of curcuminoids were represented in orange color. Hydrogen bonds were highlighted by the lines in yellow-green color, and amino acid residues which make hydrogen bonding with curcuminoids were underlined. Dotted circles indicate dissociation of amino acid residues. Right-blue, green, pink and white letters represent basic, aromatic, hydrophilic and hydrophobic amino acid residues, respectively.

Using a Glide Score top-level dissociation state of site I and II for each curcuminoid, a docking model was then prepared for each site. Figure 7 shows the docking pose for various curcuminoids and the interacting amino acid residues for site I. As is evident from the figure, the docking poses of curcuminoids at site I were similar. In addition, all curcuminoids form five hydrogen bonds in site I. The stacking interactions between the aromatic ring of Trp214 and Phe211 with curcuminoids were also a common feature for the three curcuminoids. The hydrophobic interactions with aromatic and hydrophobic amino acid residues were also similar among the curcuminoids. These results suggest that curcuminoids bind site I in a similar manner and binding affinity.

**Figure 8:**
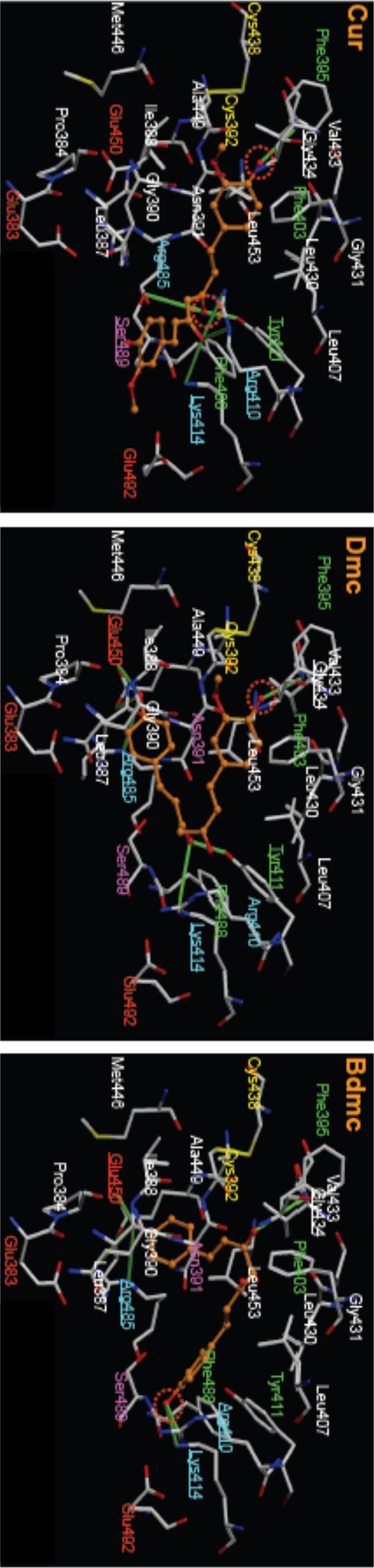
Molecular docking of curcuminoids in site II of HSA. The optimized docking poses of curcuminoids were represented in orange color. Hydrogen bonds were highlighted by the lines in yellow-green color, and amino acid residues which make hydrogen bonding with curcuminoids were underlined. Dotted circles indicate dissociation of amino acid residues. Red, right-blue, green, pink and white letters represent acidic, basic, aromatic, hydrophilic and hydrophobic amino acid residues, respectively. Cysteine residues and disulphide bond are shown in yellow letters and stick, respectively.

Figure 8 shows the docking poses of curcuminoids for site II. In contrast to site I, the docking pose of each curcuminoid at site II was different, except for the hydrogen bond with Gly434. For example, in within 4 angstroms radius from where the curcuminoids are bound, the number of sites expected to form a hydrogen bond for Cur is six locations (Gly434, Tyr411, Arg410 (two places), Lys414, Ser389), for Dmc is also six locations (Gly434, Tyr411, Lys414, Arg485, Glu450 (2 places)), and for Bdmc seven locations (Gly434, Arg410, Lys414, Arg485 (2 places), Glu450 (2 places)). In addition, Bdmc and Cur were found to be likely to form ionic bonds with Arg410 and Lys414.

**Figure 9:**
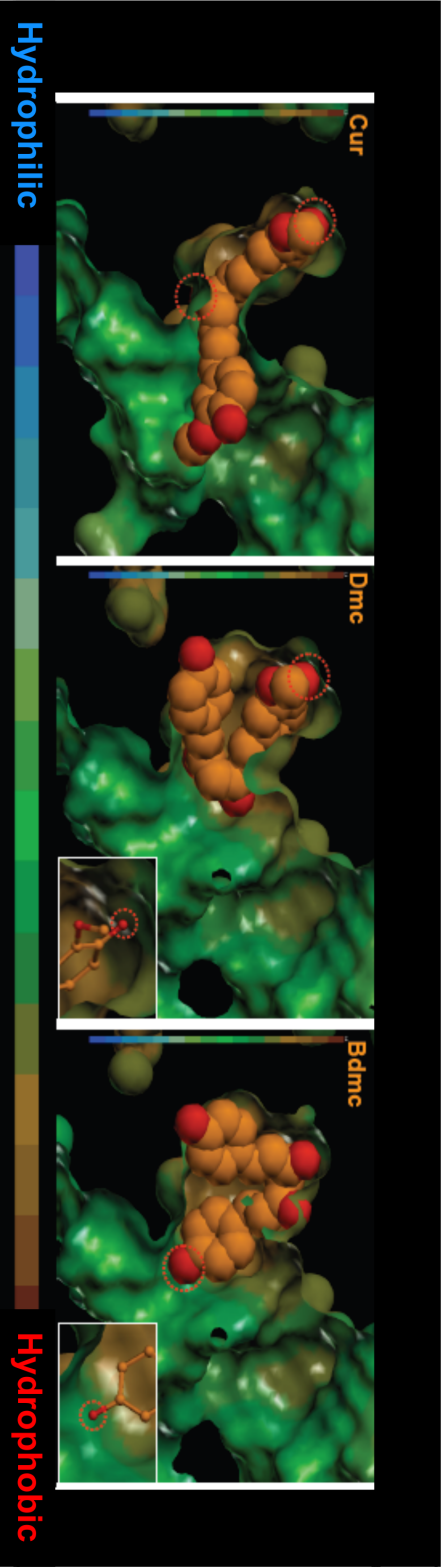
Docking poses of curcuminoids in site II of HSA, and surface charge of site II. The optimized docking poses of curcuminoids were represented in orange and red balls. Dotted circles indicate the dissociation of amino acid residues. Surface chargings were depicted by the colors shown in the level bar (blue; negatively charged, red; positively charged).

**Figure 10:**
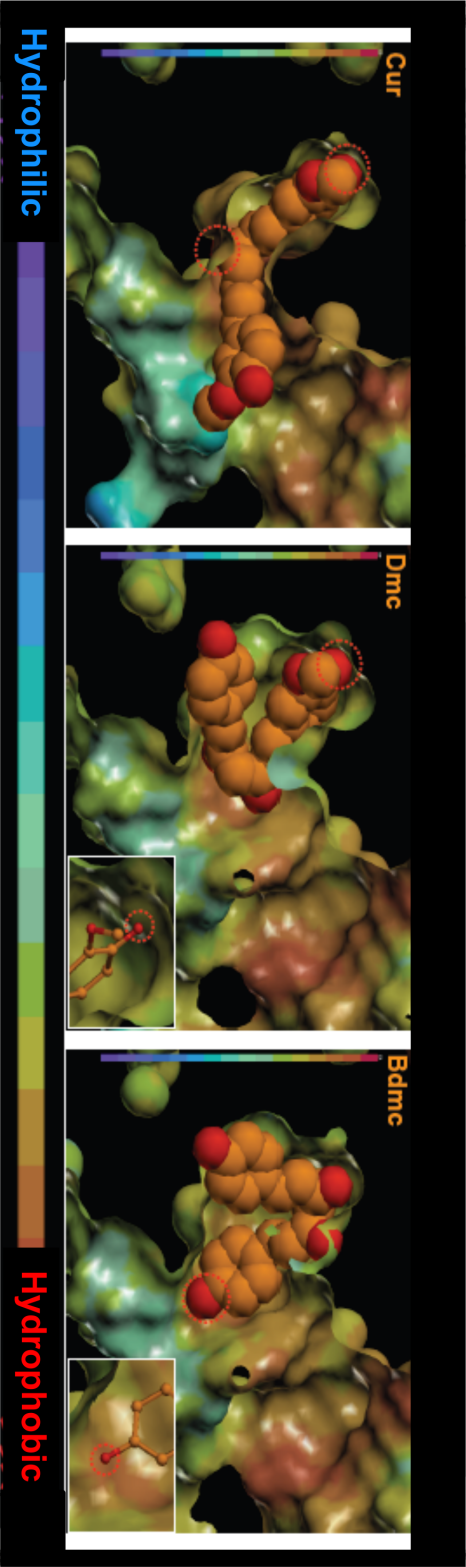
Docking poses of curcuminoids in site II of HSA, and surface hydrophobisity of site II. The optimized docking poses of curcuminoids were represented in orange and red balls. Dotted circles indicate the dissociation of amino acid residues. Surface hydrophobisities were depicted by the colors shown in the level bar (blue; hydrophilic, red; hydrophobic).

Next, the degree of surface hydrophobicity or charge state of site II was identified, the relationship between this and the docking pose of curcuminoids were then examined. As shown in Figure 9, the docking poses of Dmc and Bdmc are curled, mostly present in the hydrophobic regions. On the other hand, the docking pose of Cur is not so extensively curled, and extends across the hydrophilic region. These results suggest that the contribution of hydrophobic interactions for site II is likely to be diminished in the order of Bdmc> Dmc> Cur. Furthermore, we compared the site of localized negatively charged dissociable group of Bdmc and Dmc, and found that Bdmc tends to bind to an environment that is more electrostatically positive (Figure 10).

## Discussion

Curcuminoids have been found to possess anti-tumor and anti-Alzheimer's effects as well as a variety of biological activities [2], [3], [28], [29]. Before curcuminoids can be used clinically, further studies will be needed, including gathering information for predicting treatment effects, drug pharmacokinetics or drug interactions when used in combination with other therapeutic agents. Hence, it is essential to elucidate the full picture of the interaction with serum proteins, in particular HSA. Unfortunately, although studies on the pharmacological effects of curcuminoids have been reported, most studies on the interaction with HSA have been limited to Cur only.

In the present study, the binding properties of the curcuminoids were examined by the spectroscopic methods only, without measuring directly the unbound concentration of curcuminoids. This is because curcuminoids is readily hydrolyzed in the neutral-alkaline pH range [30]. In fact, the free concentration of Cur reported so far were obtained from indirect assessments, and no direct measurement has been performed based on binding experiments [15], [31], [32]. In addition, the CD measurements were performed at pH 9.0 in this study because no induced CD spectrum could be observed for curcuminoids at pH 7.4 [16]. Zsila et al. previously reported a distinct feature of the induced CD of Cur-HSA complex at different pH values and based on a detailed analysis of this induced CD, they concluded that Cur binds to HSA in a twisted structure (keto form) at pH 9.0 but binds to HSA in a planar structure (enol form) at pH 7.4 [16]. As shown in Figure 3, like Cur, Dmc and Bdmc also exhibit similar pH dependent induced CD spectra in the presence of HSA, suggesting that different forms of Dmc and Bdmc bind to HSA at these pHs, as mentioned above. However, since curcuminoids bind to the same binding site on HSA at pH 9.0 and pH 7.4, and that pH change does not affect the binding displacement of curcuminoids, interpretation of the binding site data obtained at pH 9.0 in relation to the physiological conditions (pH 7.4) should not be a problem.

### Identification of Curcuminoids Binding Sites on HSA

In this study, all of the curcuminoids examined caused induced-CD and fluorescence quenching of residue Trp214 in a concentration-dependent manner, suggesting that Bdmc and Dmc, like Cur, also bind to HSA. Fluorescence quenching by the addition of curcuminoids is considered to be due to energy transfer between the indole ring of Trp214 and the curcuminoids molecule. Since the only Trp residue of HSA is in the vicinity of site I, indicating that curcuminoids may bind to site I. This is consistent with data reported by Zsila [31]. On the other hand, from the displacement experiments, two binding sites for curcuminoids may exist. This is a seemingly contradictory result with the number of binding sites for curcuminoids calculated from the fluorescence quenching experiment (n = 1). A possible reason for this is that there is a curcuminoid molecule that binds to HSA but cannot quench the fluorescence derived from the Trp residue in the fluorescence quenching experiment. In fact, no intrinsic fluorescence quenching phenomenon from the ligand binding to site I of HSA was observed for site II-selective ligands such as IBF, CAP and DZP (data not shown). Therefore, the findings obtained in the fluorescence quenching experiments reflect the binding affinity of curcuminoids for site I.

In order to identify the binding sites of curcuminoids on HSA, fluorescence and CD spectra displacement experiments were performed. In the fluorescent probe displacement experiments performed at pH 7.4 and pH 9.0, all curcuminoids displaced the site I marker, WF. Curcuminoids also caused a displacement of the site II marker, DNSS, in a concentration-dependent manner. It is interesting to note that the extent of displacement of DNSS (or the binding constant to site II) varies among the curcuminoids, in the decreasing order Bdmc> Dmc> Cur. Similar results were obtained in the CD displacement experiment at pH 9.0 where the intensity of curcuminoids induced CD spectrum was reduced significantly in the presence of the site I and II markers. These results strongly suggested that curcuminoids bind to both sites I and site II.

### Binding Characteristics of Curcuminoids to Site I

Cur was reported to bind to site I by Zsila et al. [31] and Mandeville et al. [32]. Our results indicate that the other curcuminoids (Dmc and Bdmc) also bind to site I. We previously reported that site I is not a simple binding region but is rather complex and is comprised of three subsites, namely, Ia, Ib and Ic [22]. Of the site I markers used in this study, WF binds to subsite Ia, PBZ binds to subsites Ia ∼ Ib, and IDP binds to subsites Ia ∼ Ic [22]. Ghuman et al. also determined the locations of the binding regions of WF, PB and IDP in site I based on crystallographic studies [33]. WF, PB and IDP all bind to the main cavity of site I, which is composed of two apolar chambers in which Tyr150, His242, Arg257 or Arg218, Arg222 are positioned. WF accesses one of the chambers (where Tyr150, His242 and Arg257 are located), whereas PB accesses both chambers. IDP accesses not only both chambers in the main cavity, but a distinct sub-chamber separate from the main cavity which is extended to residues from subdomains IIB and IIIA. From the degree of displacement of these markers, it can be concluded that curcuminoids bind to a region of site I that is also bound by WF, PBZ but not IDP. This suggests that curcuminoids potentially bind to mainly PBZ binding region of site I, which may correspond to subsites Ia ∼ Ib. In contrast, IDP showed a slight potentiation of the induced Cotton effect of curcuminoids. Taking this phenomenon into consideration it is possible that curcuminoids and IDP both bind within site I with the aromatic ring motility of IDP immobilized by curcuminoids. Kragh-Hansen reported that site I is a large and flexible region based on a diversity of interaction ligands and that it is capable of accommodating more than one of them at a time [34]. Furthermore, crystallographic data reported by Ghuman et al. showed that significant room exists for different compounds to occupy different parts of site I, and the co-binding of indomethacin and azapropazone or phenylbutazone by shifting their position with respect to each other within site I is likely to occur [33]. Although the size of molecules that co-bind this site was not mentioned in their studies, site I may accommodate two even larger molecules, such as iodipamide and curcuminoids.

The conclusion that curcuminoids binds to site I was also supported by the reduced binding of curcuminoids to the W214A and R218A mutants, because Trp214 and Arg218 are known to be involved in the binding of site I ligands [33]. Since the degree of binding reduction to mutants for all curcuminoids were similar, the methoxy groups of curcuminoids do not appear to play a significant role in site I binding. The aromatic ring of the phenolic group that exists in all curcuminoids may have hydrophobic interaction with the indole ring1 of Trp214 while the phenolic hydroxyl group may have electrostatic interactions with Arg218. Mohammadi et al. also suggested that the phenolic hydroxyl group of Cur plays an important role in the binding process [35]. Similar structure-activity-relationships have been observed in the biological activities of curcuminoids. Kunwar et al. and Priyadarsini et al. compared the antioxidant activity of Cur and dimethoxycurcumin, and concluded that the phenolic hydroxyl group of Cur plays an important role in its anti-oxidant activity [36], [37]. Furthermore, Santosh K et al. reported that the suppression of proliferation of various tumor cell lines by Cur, Dmc, Bdmc was found to be comparable [38], indicating that the methoxy groups play minimum role in the growth-modulatory effects of Cur.

### Binding Characteristics of Curcuminoids to Site II

All curcuminoids displaced site II markers, with greater extent by IBF and CAP than DZP. Maruyama et al. reported that although the DZP binding region within site II overlaps with that of NSAIDs such as mefenamic acid and flufenamic acid, they do not match exactly [39]. In addition, Watanabe et al. reported that for site II ligands with a carboxylic acid moiety, electrostatic interaction with the guanidino group of Arg410 is important for the binding, except for the binding of DZP [20]. For ligands that bind to the same site II, different amino acid residues are involved in the binding of a ligand, and each ligand may bind in different modes. Thus, the differences in binding regions or binding modes of DZP and IBF or CAP should be considered in explaining the smaller degree of displacement by DZP. Since site II is a smaller or narrower site than site I, and, to date, no one has succeeded in dividing this site into different binding regions [40], the smaller degree of displacement may be due to differences in the binding mode between DZP and IBF or CAP. Interestingly, the degree of displacement by IBF and CAP is in the order of Bdmc> Dmc> Cur even though the binding constant of curcuminoids for site II are in the same order. This suggests that the binding mode of each of the three curcuminoids in site II is different.

We previously reported that MCO has an impact on the microenvironment of site II of HSA, causing a decrease in ligand binding [41]. In this study, MCO caused a significant decrease in the binding of curcuminoids to site II with the largest impact on Bdmc, followed by Dmc, then Cur. This result was consistent with the results of the displacement experiments described above.

Also, Tyr411 and Arg410 that play an important role in the ligand-binding of site II [20], [33] were found to also involve in curcuminoids binding, as R410A and Y411A mutants showed a significantly reduced binding for curcuminoids. From this observation, hydrogen bonding and hydrophobic interactions with Tyr411 and electrostatic interactions with Arg410 may play an important role in the binding of curcuminoids to site II. It is also possible that hydrophobic or Van der Waals interactions between these residues may be necessary for binding, in view of the drastic effect caused by the mutation into alanine residues. In addition, since the degree of binding was decreased in the same order of Bdmc> Dmc> Cur, as the displacement experiment results, this strongly suggests that, in the absence of the phenyl methoxy group interaction with the hydrophobic pocket or the interaction with Arg410 and Tyr411, a decrease in the binding affinity to site II will result. Structurally speaking, site II is less flexible than site I [24], the molecular size of Bdmc makes it easier to fit into site II than Cur. Similar relationship between curcuminoids was found by Ahmed T et al. that curcuminoids rescue long-term potentiation impaired by amyloid peptide in rat hippocampal slices in the order of Bdmc > Dmc > Cur [42].

### Investigation of the Binding Sites with Modeling

The results obtained from docking simulations of curcuminoids in site I and site II of HSA were in agreement with the results of the displacement experiments. In the docking model of site I, the docking pose of each curcuminoid and the environment of molecular interactions such as hydrogen bonding were similar. These results confirmed that the binding affinity among the curcuminoids for site I is similar.

In contrast, the docking poses of curcuminoids for site II site were clearly different. Bdmc and Dmc took a rounded form when binding to a region in site II that was positively charged and hydrophobic, whereas Cur was in a more extended form binding to a region that was relatively negatively charged and hydrophilic. Therefore, the hydrophobic or electrostatic interaction of Cur with site II is weaker than Dmc and Bdmc. In addition, Bdmc is more suitable for evaluating electrostatic or hydrophobic interactions than Dmc with the surrounding environment and with the negatively charged dissociated oxygen atoms. Bdmc forms stronger hydrogen bonds than Dmc. Having cross interpreted these results, the binding affinity of curcuminoids for site II diminished in the order of Bdmc> Dmc> Cur, which matched and agreed very well with the results of binding and displacement experiments. In order to further verify these findings, Bdmc was docked in site II with a methoxy group introduced into two locations of the molecule similar to those in Cur. The methoxy groups were found to be in obstructive contact with the pocket region of site II, making it difficult for Bdmc to fit in properly at the binding region. Hence, the reason for why Cur binds in an extended form to site II was the two methoxy groups of Cur introduced steric hindrance that prevented it from binding to site II in the same manner as Dmc and Bdmc. In addition, the discrepancy in the binding affinity to site II was also due to different amino acid residues involved in the binding interaction of curcuminoids.

## Conclusions

In this study, Dmc and Bdmc were found to also bind to both site I and site II of HSA. No difference among curcuminoids when binding to site I was found, but binding at site II differs depending on the number of methoxy groups present on the phenyl group of the curcuminoids.
